# Effects of multisession prefrontal cortex tDCS or taVNS on stress, perceived stress and sleep quality: a double-blind, randomized controlled study

**DOI:** 10.3389/fpsyg.2024.1343413

**Published:** 2024-09-13

**Authors:** Laya Dalila dos Reis, Laura Pereira Generoso, Gabrielly Santos Pereira, João Paulo da Silva Teixeira Barú, Natalie Lange Candido, Maria Gabriela Maziero Capello, Renato Ortolani Marcondes de Castro, Edvaldo José Rodrigues Cardoso, Robson Dias Scoz, Luciano Maia Alves Ferreira, Marcelo Lourenço da Silva, Josie Resende Torres da Silva

**Affiliations:** ^1^Laboratory of Neuroscience, Neuromodulation and Study of Pain (LANNED), Federal University of Alfenas (UNIFAL-MG), Alfenas, Minas Gerais, Brazil; ^2^Neuromodulation and Pain Unit (NeuroPain), Egas Moniz Interdisciplinary Research Center (CiiEM), Almada, Portugal

**Keywords:** chronic stress, transcranial direct current stimulation, transcutaneous auricular vagus nerve stimulation, sleep quality, neuromodulation

## Abstract

**Introduction:**

Chronic stress is a condition characterized by prolonged stimulation, leading to mental and physical weakness. It can have detrimental effects on individuals’ mental health and cognitive function, potentially causing various health issues. This article explores the potential of non-invasive neuromodulation techniques, specifically transcranial direct current stimulation (tDCS) and transcutaneous auricular vagus nerve stimulation (taVNS), in managing chronic stress and improving sleep quality.

**Methods:**

The study conducted a randomized, double-blinded, controlled trial with participants experiencing chronic stress. In total, 100 participants were randomly assigned to one of four conditions: the anodal tDCS group (*n* = 50), the sham tDCS group (*n* = 50), the taVNS group (*n* = 50), or the sham taVNS group (*n* = 50). Within each condition, participants received five sessions of either active treatment or sham treatment, with 20 min of tDCS over the dorsolateral prefrontal cortex (2 mA) for the tDCS groups, or taVNS on the left ear (20 Hz) for the taVNS groups. At baseline, post-intervention, and 4 weeks thereafter, we evaluated stress using the Lipp’s Inventory of Stress Symptoms for Adults (LSSI), perceived stress through the Perceived Stress Scale (PSS-10), and sleep quality via the Pittsburgh Sleep Quality Index (PSQI).

**Results:**

The tDCS and taVNS interventions resulted in reduced stress levels, improved sleep quality, and enhanced perception of stress.

**Discussion:**

These findings suggest that tDCS and taVNS hold promise as effective treatments for chronic stress, offering a safe and accessible approach to improving individuals’ wellbeing and overall quality of life.

**Clinical trial registration:**

https://ensaiosclinicos.gov.br/rg/RBR-2ww2ts8, identifier UTN: U1111-1296-1810; Brazilian Registry of Clinical Trials (REBEC) RBR-2ww2ts8.

## 1 Introduction

Chronic stress occurs when individuals experience prolonged stimulation, leading to both mental and physical weakness (Liu et al., 2023). Typically, chronic stress can arise from exposure to stressors for a duration exceeding 30 days or from stressors that have enduring and lasting effects on individuals for more than 30 days (Stoney et al., 1999). Is a lasting condition that has the potential to impact individuals’ mental health and cognitive function (Koenen et al., 2017). Individuals experiencing multiple psychological stressors due to chronic stress may face a range of health issues, including anxiety, insomnia, chronic pain, hypertension, and weakened immune system (Pruett, 2003; Lindfors et al., 2017; Zhang et al., 2018), which can ultimately lead to the development of life-threatening diseases like heart disease and depression (Milner et al., 2017).

The development of advanced neuroimaging modalities has advanced our understanding of brain function during the interaction among various stress contributors. Chronic stress induces significant changes in the sympathetic neural system related to behavioral suppression. Due to the neural complex, there are connections between the prefrontal cortex (PFC) and the limbic system. The PFC is sufficiently resilient to balance dopamine levels in the limbic system (Alyan et al., 2021).

In a previous study, it was found that the application of transcranial direct current stimulation (tDCS) to the left-side dorsolateral prefrontal cortex (DLPFC) resulted in a reduction of the reported negative effects caused by daily stressors (Austin et al., 2016). TDCS modulates subthreshold cortical excitability and plasticity by polarizing nerve tissue using low-intensity electrical currents (1–2.5 mA) administered over the scalp. The effects of tDCS are believed to involve an increase in cortical excitability with anodal modulation and a decrease with cathodal modulation (Stagg et al., 2018; Chan et al., 2021) reducing stress symptoms (Kadam et al., 2001; Smits et al., 2021). However, the neurophysiological effects of single-session tDCS are typically transient and tend to diminish within a few hours and not consistently produce effective modulation of stress regulation (Smits et al., 2023).

Transcutaneous auricular vagus nerve stimulation (taVNS) involves the noninvasive neuromodulation of peripheral nerves located beneath the skin of the ear. In recent times, taVNS has garnered significant scientific attention due to its potential for facilitating a bottom-up approach to regulating stress and sleep, exerting influence from subcortical to cortical structures (Li et al., 2020; Agarwal et al., 2023; Zhao et al., 2023). While the specific mechanisms underlying the effects of taVNS in stress are not yet fully understood, it holds promising potential for intervening at the neurobiological level of these disorders (Bremner et al., 2020; Zhao et al., 2023). Previous studies have demonstrated that the timing of activation in brain structures, such as the locus coeruleus, can be influenced by varying pulse frequencies of taVNS (Höper et al., 2022).

In our randomized, double-blinded, controlled trial, we aimed to evaluate the impact of repeated DLPFC tDCS or taVNS sessions on managing chronic stress. Our primary objective was to investigate whether the multisession protocol of tDCS or taVNS could effectively alleviate stress and subsequently enhance sleep quality in individuals experiencing chronic stress. In this study, we hypothesized that both tDCS and taVNS would demonstrate efficacy in reducing stress levels and improving sleep quality among participants with chronic stress. Specifically, we posited that active tDCS, when targeting specific brain regions associated with stress regulation, would lead to a greater reduction in perceived stress levels compared to sham tDCS. Similarly, we anticipated that taVNS, through its stimulation of the vagus nerve, would result in improvements in both subjective stress perception and sleep quality.

## 2 Materials and methods

### 2.1 Participants

The present study recruited a total of 175 individuals from municipal government healthcare (Alfenas-MG, Brazil), and the treatments provided were entirely through the Unified Health System (SUS). We used the Lipp’s Inventory of Stress Symptoms for Adults (LSSI) to select participants. Participants meeting the inclusion criteria scored above 4 on the LSSI, indicating a moderate to high level of stress symptoms (Pawlina et al., 2015), and above 5 on the Pittsburgh Sleep Quality Index (PSQI), indicating significant sleep disturbances and poorer sleep quality (Bertolazi et al., 2011). These thresholds qualified them as suitable candidates for assessing the effects of interventions on stress management. In total, 100 participants were included in our study. Participants were randomly assigned following simple randomization procedures (computerized random numbers) to 1 of 4 treatment groups; the anodal tDCS group (*n* = 25; 11 females); or the sham tDCS group (*n* = 25; 14 females); taVNS group (*n* = 25; 15 females); or the sham taVNS group (*n* = 25; 10 females).

Creating four groups, rather than two, covering both active and sham conditions for each technique, allows for a more thorough assessment of the interventions. It helps discern specific effects from potential placebos, controls for confounding variables, and facilitates comparisons between active and sham conditions within each technique. We used GPower 3.1 for *post hoc* power analyses, and the results showed that when the effect size is set to 0.2, our sample size (*n* = 100, 25 per group) holds a power (1 − β) of 0.85. We screened all participants for individuals with normal or corrected-to-normal vision, those who are right-handed, assessed using a self-report questionnaire where participants indicated their dominant hand, and who have no history of severe psychological disorders, assessed using either self-report measures and standardized diagnostic instruments administered by trained psychologist (LR). All participants were requested to refrain from consuming any kind of substances or taking medications that may potentially affect their focus for a week leading up to the experiment. Individuals with a history of dizziness or seizures, pregnancy, and signs of severity were excluded. Exclusion criteria also included a diagnosis of bipolar mood disorder with depressive, manic, or hypomanic symptoms in the past year; schizophrenia or other psychotic disorders; autism; substance dependence; and a diagnosis of epilepsy or use of anticonvulsant medications. All written consent were signed before participating in the study. This study was approved by the Federal University of Alfenas Ethics Committee (CAAE 51925921.9.0000.5142) and registered in the Brazilian Registry of Clinical Trials (ReBEC) number RBR-2ww2ts8.

### 2.2 Instruments

#### 2.2.1 Lipp’s Inventory of Stress Symptoms for Adults

A semi-structured interview was conducted to collect anamnesis data regarding the participants’ sociodemographic profile. The LSSI was used to evaluate stress indicators (Lipp and Guevara, 1994) and adapted to Brazilian Portuguese by Anunciação et al. (2022). The purpose of this inventory is to identify stress patterns and diagnose stress in adults, categorizing them into stages of alertness, resistance, near-exhaustion, and exhaustion. It is based on a four-phase model and emphasizes somatic and psychologically related symptoms of stress. The sample group includes individuals aged 20–50 of both genders, and the application of the inventory takes approximately 15 min. The maximum score achievable on the questionnaire is 40. Scores above 4 on the LSSI indicates a moderate to high level of stress symptoms. The Cronbach’s α of the original LSSI was 0.93 (Anunciação et al., 2022). In this study, it was 0.94.

#### 2.2.2 Perceived Stress Scale

Additionally, the Perceived Stress Scale (PSS-10) was administered (Taylor, 2015; Nielsen et al., 2016). The PSS-10 questionnaire comprises 10 items that assess subjective feelings associated with daily challenges, personal experiences, coping mechanisms, and behaviors within the past month. This scale serves as a straightforward and dependable assessment tool, applicable in both clinical and research settings. The total score is obtained by summing the responses to the 10 individual questions. The maximum score achievable on the questionnaire is 40. Scores ranging from 0 to 13 indicate low stress exposure, while scores above 20–22 suggest high levels of stress exposure. The version used in this study was translated and adapted to Brazilian Portuguese by Luft et al. (2007). The Cronbach’s α of the Brazilian Portuguese version of the PSS-10 was 0.82 (Luft et al., 2007). In this study, it was 0.94.

#### 2.2.3 Pittsburgh Sleep Quality Index

For sleep quality assessment, the PSQI was employed. This tool is used to evaluate sleep quality and possible disturbances in the previous month. It was developed by Buysse et al. (1989) and validated in the adult Brazilian population by Bertolazi et al. (2011). The PSQI consists of 19 self-administered items to assess sleep quality and patterns over the previous month. These items are used to derive seven subscales, including sleep quality, sleep latency, sleep duration, habitual sleep efficiency, sleep disturbances, daytime dysfunction, and use of sleep medication. Subscale scores (equally weighted from 0 to 3) are summed to obtain the overall PSQI score (ranging from 0 to 21), with higher scores (>5) indicating significant sleep disturbances and poorer sleep quality. The Cronbach’s α of the original PSQI was 0.83 (Buysse et al., 1989). In this study, it was 0.85.

### 2.3 Interventions

#### 2.3.1 tDCS parameters

A Low-Intensity transcranial DC Stimulator (Microestim Foco Research NKL, Brusque, Brazil) was used in the present protocol. Based on the international EEG 10–20 system and previous research (Austin et al., 2016; Dubreuil-Vall et al., 2019), the neuromodulation were conducted using 7 cm × 5 cm (35 cm^2^) electrodes, with the anode positioned on the left DLPFC at F3 and the cathode on the right DLPFC at F4. Previous studies have indicated that anodal placement on the left with the cathode in a contralateral homologous region stimulation may have a potentially superior effect compared to the anode on the right hemisphere (Dubreuil-Vall et al., 2021). During the neuromodulation, one investigator, who was aware of the participants’ group randomization, set up the stimulator according to the protocol for both the sham and active anodal-tDCS conditions. This investigator was not involved in any other data collection procedures. Participants in the active anodal tDCS group received a 2-mA stimulation for 20 min daily over 5 consecutive days. The ramp-up and ramp-down phases lasted for 30 s at the beginning and end of the neuromodulation. In the sham tDCS group, the electrodes were placed in the same locations as the active anodal tDCS group to mimic the potential tingling sensation associated with active stimulation, but no sustained effects on cortical activity were induced. The currents were only applied during the 30-s ramp-up and ramp-down phases at the beginning and end of the 20-min sham-neuromodulation period.

#### 2.3.2 taVNS parameters

An electrical stimulator (EL608 Digital Connect NKL, Brusque, Brazil) was used in the present protocol. The electrodes were positioned on the upper concha of the left ear with gel to ensure better distribution of the current (Staley et al., 2020). Previous studies have indicated that left ear stimulation may have a potentially superior effect compared to the right ear (Badran et al., 2019). During the neuromodulation, one investigator, who was aware of the participants’ group randomization, set up the stimulator according to the protocol for both the sham and active taVNS conditions. This investigator was not involved in any other data collection procedures. Participants in the active taVNS group received a 20 Hz neuromodulation for 20 min daily over 5 consecutive days. In the sham taVNS group, the electrodes were placed in the same locations as the active taVNS group to mimic the potential tingling sensation associated with active neuromodulation. The frequency as set to 0 Hz for 20 min daily over 5 consecutive days.

### 2.4 Experimental procedures

**FIGURE 1:**
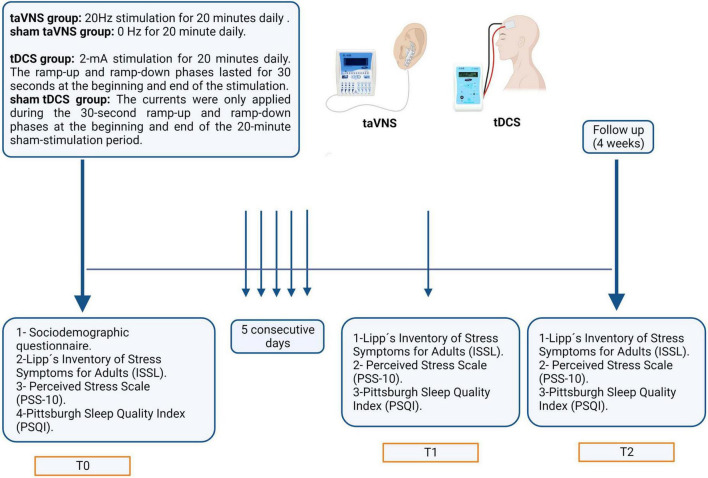
The timeline of the data collection process over 28 days. On the initial day (T0), participants completed sociodemographic profiles and pre-tests for the LSSI, PSS-10, and PSQI. Over the subsequent 5 days, participants in the active anodal tDCS or taVNS groups received daily 20-min sessions of neuromodulation, while sham groups received sham treatment for the same duration. On the seventh day (T1) and 4 weeks post-intervention (follow-up, T2), all participants completed post-tests for the LSSI, PSS-10, and PSQI.

The design of the double-blind randomized controlled trial was conducted to ensure that participants were randomly assigned to either the Active or Sham groups, and that the researcher performing the experiment and participants were unaware of the allocation. As shown in Figure 1, the data collection process took 28 days to complete. On the first day (T0), participants completed the sociodemographic profile and the pre-test of the LSSI, the PSS-10, and the PSQI. For the subsequent 5 days, participants in the active anodal tDCS or taVNS groups received 20 min of neuromodulation daily, while those in the sham groups received sham neuromodulation for the same duration. On the seventh day (T1) and 4 weeks after the intervention (follow-up, T2), all participants completed the post-test of the LSSI, the PSS-10, and the PSQI.

### 2.5 Potential side effects

Potential side effects of the procedure are minimal, and there have been no reports of significant adverse events associated with low-current procedures similar to those used in this study. Possible side effects of low-current tDCS may include localized itching or tingling sensations on the scalp where the electrode was placed, and rarely, headache or fatigue (Lefaucheur et al., 2017). For taVNS the possible side effects may include ear pain, dizziness, skin redness, and headache (Kim et al., 2022). If any of these side effects occur, the participant will be closely monitored. If the symptoms persist for more than 1 hour, the participant will be referred to a medical professional for further evaluation. Instances of discontinuation or withdrawal from the study will be recorded in the study database.

### 2.6 Statistical analysis

For statistical analysis, the Statistical Package for the Social Sciences (SPSS) software (IBM Corp., Chicago, IL, USA), version 20.0, was used. Initially, all data sets from the sample were tested for normality using the Kolmogorov–Smirnov test. After this analysis, a one-way analysis of variance (ANOVA) test was conducted if the sample followed a normal distribution. If the data did not meet the normality assumption, the Kruskal–Wallis test was applied. Regarding the independent variables, if the normality criterion was met, a Student’s *t*-test was conducted. If the criterion was not met, the Mann–Whitney U test was used for intergroup comparisons. For both tests, a significance level of 5% was considered.

## 3 Results

**FIGURE 2:**
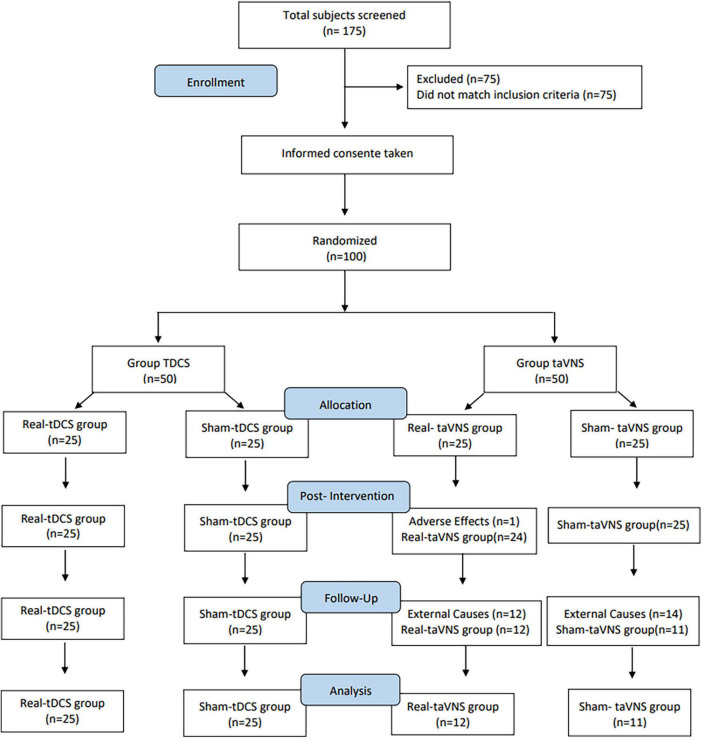
CONSORT diagram. Flowchart of participants disposition throughout the study.

One hundred, seventh five individuals were screened (Figure 2). One hundred aged between 20 and 59 years (50 females and 50 males; mean age 37.2 years) were included in the study. One participant from the active taVNS group dropped out of the study during the intervention because adverse effects (headache – received three taVNS sessions before dropping out). Twenty-six individuals were lost to follow-up (external causes; 12 on active taVNS and 14 on sham taVNS groups). Data from the participants who dropped out were excluded from all analyses. The authors conducted a comprehensive examination of the dataset to identify outlier participants across the variables under investigation. However, no outliers were found upon thorough analysis. The demographic and general clinical characteristics of the participants are summarized in Table 1.

**TABLE 1:** Study patient demographics.

Characteristics	Total full sample	Active tDCS	Sham tDCS	Active taVNS	Sham taVNS
**Sex, no.**					
Female	50	13	12	12	13
Male	50	12	13	13	12
**Age**					
20–29	12	4	6	2	0
30–39	24	6	9	4	5
40–49	16	5	4	3	4
50–59	23	10	6	3	4
**Marital status**					
Single	22	15	17	15	15
Married/common law	40	15	16	15	14
Divorced (or legally separated)	11	5	2	2	2
**Race**					
White	41	16	15	19	11
Black	4	12	10	10	12
Brown	28	7	10	3	8
**Psychotherapy**					
Yes	15	15	12	14	13
No	56	10	13	11	12
**Physical activity**					
Yes	31	13	8	9	5
No	42	12	17	16	20
**Pharmacotherapy**					
Anxiolytic	5	1	2	1	1
Antidepressants	20	5	2	2	1
None	48	14	16	19	19

### 3.1 tDCS intervention on LSSI

**FIGURE 3:**
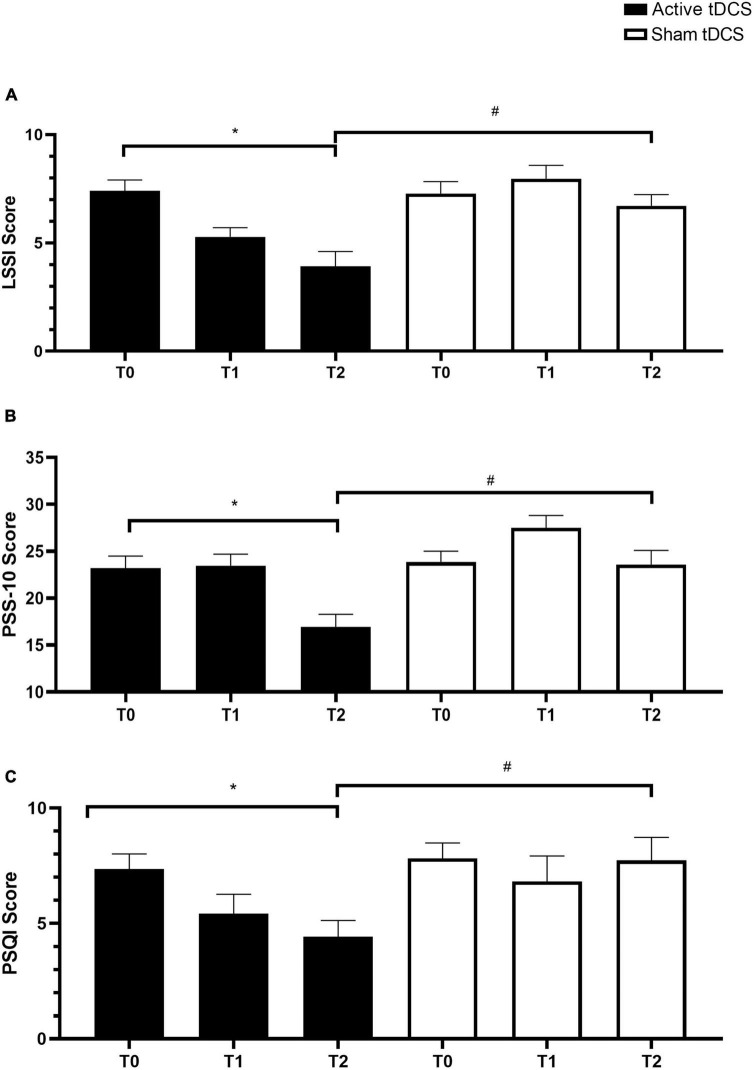
Changes in stress levels and sleep quality following tDCS intervention. **(A)** Mean scores on the LSSI questionnaire at baseline (T0), immediately post-treatment (T1), and follow-up (T2) for the active anodal tDCS and sham tDCS groups. **(B)** Mean scores on the PSS-10 questionnaire at T0, T1, and T2 for both groups. **(C)** Mean total PSQI scores at T0, T1, and T2 for both groups. Error bars represent standard error of the mean (SEM). *p < 0.05 within groups and ^#^*p* < 0.05 between groups.

Analysis of the LSSI questionnaire (Figure 3A) revealed significant differences between T1 and T0 in the active anodal tDCS group (*F*_5,143_ = 7.552, *p* < 0.001, η^2^ = 0.32), indicating a reduction in stress levels compared to baseline. Normality tests indicated that the data for this analysis were normally distributed (Shapiro–Wilk test, *p* > 0.05). This reduction was sustained at T2. In contrast, the sham treatment group showed an initial increase in stress levels at T1 compared to T0, which significantly decreased by T2, although remaining lower than the active treatment group. Regarding the comparison of T2 to T1, both the active and sham groups demonstrated a significant decrease in stress levels (*p* < 0.001). Additionally, the delta T1–T0 comparison revealed a significantly greater reduction in stress levels in the active group compared to the sham group (*p* < 0.001, Cohen’s *d* = 0.72). Similarly, the delta T2–T1 comparison showed a significantly greater decrease in stress levels in the active group compared to the sham group (*p* < 0.001, Cohen’s *d* = 0.68).

### 3.2 tDCS intervention on PSS-10

The analysis of PSS-10 scores (Figure 3B) revealed significant findings. Firstly, participants subjected to active anodal tDCS exhibited a significant reduction in perceived stress from T1 to T2 (*F*_5,119_ = 1.518, *p* < 0.001, η^2^ = 0.29). Normality tests confirmed that the data for this analysis were normally distributed (Shapiro–Wilk test, *p* > 0.05). Conversely, the sham tDCS group displayed a significant increase in stress levels immediately post-treatment at T1, followed by a slight, non-significant improvement at T2. Additionally, when comparing delta scores between T1 and T0, the active anodal tDCS group showed a significantly greater reduction in perceived stress compared to the sham tDCS group (*p* < 0.001, Cohen’s *d* = 0.74). Similarly, the delta comparison between T2 and T1 indicated a significantly greater decrease in stress levels in the active group compared to the sham group (*p* < 0.001, Cohen’s *d* = 0.71). These results underscore the efficacy of active anodal tDCS in improving the perception of stress compared to sham tDCS.

### 3.3 tDCS intervention on PSQI

Analysis of the total PSQI scores (Figure 3C) revealed significant findings. Firstly, participants receiving active tDCS exhibited a gradual and positive improvement in sleep quality from T1 to T2 (*F*_5,119_ = 6.273, *p* < 0.001, η^2^ = 0.35). Normality tests confirmed that the data for this analysis were normally distributed (Shapiro–Wilk test, *p* > 0.05). In contrast, the sham tDCS group showed no notable improvement in sleep quality. When comparing delta scores between T1 and T0, the active tDCS group displayed a significantly greater improvement in sleep quality compared to the sham tDCS group (*p* < 0.001, Cohen’s *d* = 0.80). Similarly, the delta comparison between T2 and T1 indicated a significantly greater enhancement in sleep quality in the active group compared to the sham group (*p* < 0.001, Cohen’s *d* = 0.75). These results highlight the beneficial impact of active tDCS on sleep quality compared to sham tDCS.

### 3.4 taVNS intervention on LSSI

**FIGURE 4:**
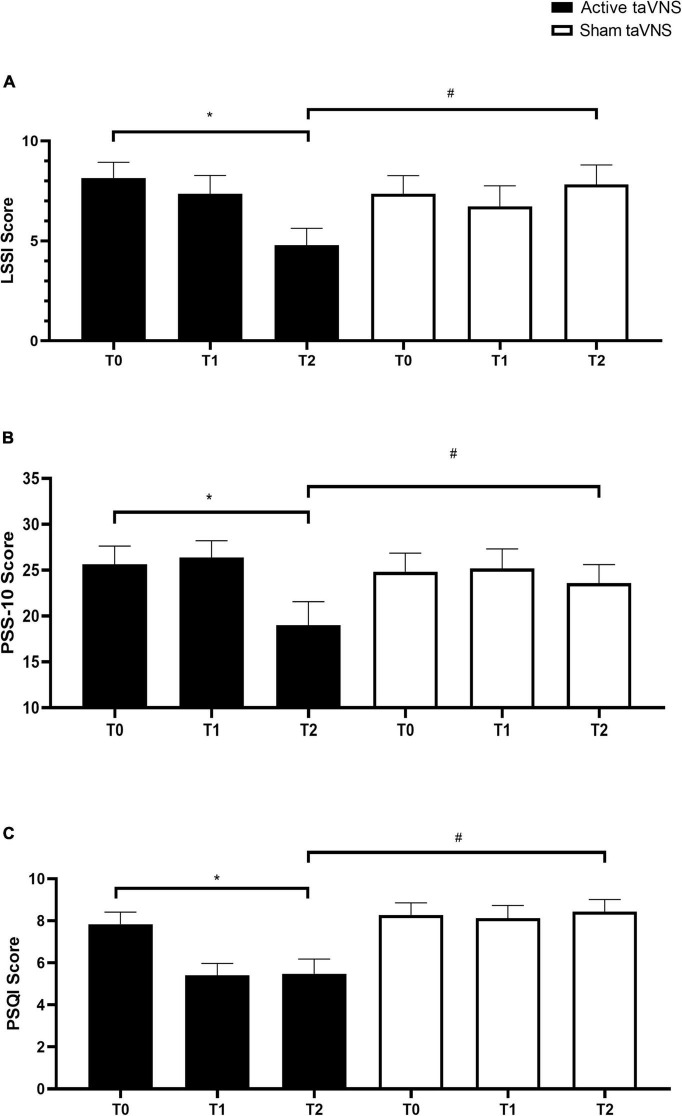
Effects of taVNS intervention on stress levels and sleep quality. **(A)** Mean scores on the LSSI questionnaire at T0, T1, and T2 for the active taVNS and sham taVNS groups. **(B)** Mean scores on the PSS-10 questionnaire at T0, T1, and T2 for both groups. **(C)** Mean total PSQI scores at T0, T1, and T2 for both groups. Error bars represent standard error of the mean (SEM). *p < 0.05 within groups and ^#^*p* < 0.05 between groups.

Analysis of the LSSI questionnaire (Figure 4A) revealed significant differences between T1 and T0 in the active taVNS group (*F*_5,69_ = 1.923, *p* < 0.001, η^2^ = 0.28), indicating a gradual regulation of stress levels over time. Normality tests indicated that the data for this analysis were normally distributed (Shapiro–Wilk test, *p* > 0.05). Comparison between T2 and T1 showed a continued reduction in stress levels in the active taVNS group, although not statistically significant. Delta comparison between T1 and T0 demonstrated a significant decrease in stress levels in the active taVNS group compared to the sham taVNS group (*p* < 0.001, Cohen’s *d* = 0.68), indicating the effectiveness of active taVNS in stress reduction. Similarly, the delta comparison between T2 and T1 showed a trend toward further stress reduction in the active taVNS group compared to the sham taVNS group, although not statistically significant.

### 3.5 taVNS intervention on PSS-10

Results from the PSS-10 questionnaire (Figure 4B) indicated a notable improvement in perceived stress levels from T1 to T2 in the active taVNS group (*F*_5,119_ = 6.273, *p* < 0.001, η^2^ = 0.26), indicating a gradual reduction in stress perception over time. Normality tests confirmed that the data for this analysis were normally distributed (Shapiro–Wilk test, *p* > 0.05). Comparison between T2 and T1 showed a further improvement in stress perception in the active taVNS group, although not statistically significant. Delta comparison between T1 and T0 demonstrated a significant decrease in perceived stress levels in the active taVNS group compared to the sham taVNS group (*p* < 0.001, Cohen’s *d* = 0.75), highlighting the efficacy of active taVNS in reducing perceived stress. Similarly, the delta comparison between T2 and T1 indicated a trend toward continued improvement in stress perception in the active taVNS group compared to the sham taVNS group, although not statistically significant.

### 3.6 taVNS intervention on PSQI

Analysis of the PSQI scores (Figure 4C) revealed significant improvements in sleep quality from T1 to T2 in the active taVNS group (*F*_5,119_ = 5.617, *p* < 0.001, η^2^ = 0.24), indicating a positive change in sleep quality over time. Normality tests confirmed that the data for this analysis were normally distributed (Shapiro–Wilk test, *p* > 0.05). No significant improvement was observed in the Sham group between T1 and T0. Delta comparison between T1 and T0 demonstrated a significant improvement in sleep quality in the active taVNS group compared to the sham taVNS group (*p* < 0.001, Cohen’s *d* = 0.70), highlighting the superiority of active taVNS in enhancing sleep quality. Similarly, the delta comparison between T2 and T1 indicated a trend toward sustained improvement in sleep quality in the active taVNS group compared to the sham taVNS group, although not statistically significant.

### 3.7 Comparison between techniques

Exploration of the comparison between tDCS and taVNS interventions revealed significant differences in their effects on stress levels, perceived stress, and sleep quality. While both interventions showed efficacy in reducing stress and improving sleep quality, the effect sizes and patterns of improvement differed between the two techniques. tDCS demonstrated larger effect sizes in reducing stress levels (Cohen’s *d* = 0.72–0.80) and enhancing sleep quality (Cohen’s *d* = 0.75–0.80) compared to taVNS (Cohen’s *d* = 0.68–0.75). Conversely, taVNS showed a more gradual but sustained improvement in stress regulation and perceived stress reduction over time. These findings suggest that while both techniques offer therapeutic benefits, their mechanisms of action and effectiveness may vary, providing valuable insights for future research and clinical applications.

## 4 Discussion

In our study, we investigated the effects of tDCS or taVNS on stress and sleep quality control in chronically stressed individuals. Based on our present results, tDCS targeting the left DLPFC or taVNS at the left ear could relieve chronic stress and improve sleep quality, which was confirmed by the decreased perceived stress (LSSI and PSS-10) and positive improvement in sleep quality (PSQI) in the post-test groups compared with the pre-test groups.

These findings are consistent with previous studies investigating the effects of tDCS and taVNS on stress and sleep. Several studies have demonstrated the effectiveness of tDCS in reducing stress and improving sleep quality. For example, a study by Fregni et al. (2006) found that repeated sessions of tDCS in patients with depression led to cognitive improvements and a reduction in perceived stress. Similarly, a randomized controlled trial showed that taVNS improved major depressive disorder symptoms, which are often associated with chronic stress (Brunoni et al., 2013; Rong et al., 2016).

Regarding sleep quality, a systematic review and meta-analysis by Liu et al. (2020) concluded that taVNS has a positive effect on sleep quality. The meta-analysis included multiple studies and showed significant improvements in various sleep parameters, including sleep duration and sleep efficiency. These findings align with our study, as we observed a significant improvement in sleep quality following tDCS and taVNS interventions. The positive changes in perceived stress and sleep quality support the notion that these non-invasive neuromodulation techniques have beneficial effects in managing chronic stress and sleep disturbances (Jiao et al., 2020).

In this study, the techniques and approach were conducted in a manner to minimize any placebo effect. Placebo, as described by Colloca (2020), refers to simulated interventions, treatments, and substances. Verbal suggestion is one of the primary methods for inducing the placebo effect. Participants in this study may have believed they were receiving the genuine treatment, as the activation of electrodes for both tDCS and taVNS were identical, and the study was blinded, including the researcher (Kaptchuk et al., 2010).

Mental disorders are among the leading causes of disability worldwide. These disorders, such as depression, anxiety, adjustment disorders, and stress-related symptoms, pose a significant occupational health problem due to their negative impact on work capacity and productivity. Mental disorders and stress-related symptoms can lead to long-term sick leave and work disability (Lai et al., 2020).

Stress and its associated symptoms can lead to a range of negative consequences for healthcare professionals, including burnout syndrome, fatigue, insomnia, anxiety, depression, obesity, cardiovascular diseases, diabetes, and psychosomatic disorders. These can affect the quality of healthcare services and patient satisfaction (Ríos-Santos et al., 2010). The findings of this study, using the PSS-10 scale, show that individuals perceived themselves as stressed, and there was an improvement in perception when treated with active tDCS, potentially enhancing the quality of services and the overall wellbeing of individuals (Lee and Ashforth, 1996).

Sleep is a crucial biological function for humans as it plays a significant role in cognitive processes, physical and mental health. Sleep quality is influenced by age, culture, environmental factors, psychological conditions, and physiological factors. Poor sleep quality is associated with higher mortality rates and increased prevalence of conditions such as diabetes, hypertension, coronary artery disease, and depression. The relationship between sleep disorders and poor sleep quality is a frequent cause of traffic and work accidents due to excessive sleepiness (Cappuccio et al., 2010; Grandner et al., 2010).

In this study, sleep quality improved gradually and significantly in individuals treated with active tDCS, indicating an improvement not only in sleep quality but also in various aspects related to sleep, thereby enhancing individuals’ overall quality of life. Chronic psychosocial stress affects the brain in a stereotypical manner, regardless of the underlying cause. Changes in the brain are not limited to exposure to extreme or life-threatening situations but can also be related to daily stress. Therefore, new proposals for stress management with low side effects and effective restoration of mental health are necessary (McEwen, 2000; Lupien et al., 2009).

Transcranial direct current stimulation has become increasingly popular as a non-invasive, well-tolerated, cost-effective, and high-yield approach. Previous studies have shown that the effects of tDCS can last up to an hour after neuromodulation. The mechanisms of action involve modifying the synaptic microenvironment, altering NMDA receptor synaptic strength, modulating GABAergic activity, and influencing excitability by modulating intracortical and corticospinal neurons. As most neurotransmitters and receptors in the brain have electrical properties, the constant electric field generated by tDCS can induce long-lasting neurochemical changes. Repeated sessions have been recommended to achieve longer-lasting effects, which have been associated with greater magnitude and duration of behavioral effects (Nitsche et al., 2008; Bikson et al., 2016).

Another intervention for stress and sleep quality improvement is vagus nerve stimulation. Studies have demonstrated the effects of taVNS on autonomic tone, cardiovascular function, and central areas of the brain involved in emotional modulation. TaVNS has shown safety and efficacy in humans, being practical to use and cost-effective, thus representing a promising therapeutic modality in the field of neuromodulation in clinical psychiatry (Farmer et al., 2021).

In this study, only one adverse event was reported, severe insomnia during the 2 days of treatment, leading to its discontinuation. Adverse effects of vagus nerve stimulation are mainly related to the stimulation itself, experienced for very short intermittent periods and in invasive neuromodulation. Common adverse effects include voice alteration, cough, dyspnea, dysphagia, cervical pain, or paresthesia. Stimulation parameters can be adjusted to make adverse effects more tolerable.

The absence of significant results between T0 and T1 for both tDCS and taVNS interventions suggests that immediate changes in stress levels and sleep quality may not be expected following the initial treatment sessions. Instead, these interventions may require multiple sessions or a longer duration to produce noticeable effects, indicating a potential cumulative or gradual alteration in neural activity and neurochemical processes (Stagg et al., 2013). However, the lack of significant results during this initial phase does not negate the possibility of long-term benefits, as observed improvements in stress and sleep quality could be indicative of sustained effects that manifest over time with continued intervention. Furthermore, the inclusion of a T3 assessment with a similar interval as T1–T2 would provide valuable insights into the durability and stability of the treatment effects, offering a more comprehensive understanding of the long-term efficacy of tDCS and taVNS in managing chronic stress and sleep disturbances (Su et al., 2024).

Despite the significant findings and contributions of this study, several limitations should be acknowledged. Firstly, the sample size was relatively small, limiting the generalizability of the results. Future studies with larger sample sizes are warranted to validate the findings and explore potential moderators of treatment effects. Secondly, the study duration was relatively short-term, and longer follow-up periods are needed to assess the durability of treatment effects and potential relapse rates. Additionally, while efforts were made to minimize placebo effects, the possibility of placebo responses cannot be entirely ruled out. Further research incorporating rigorous placebo-controlled designs and objective outcome measures is needed to elucidate the true efficacy of tDCS and taVNS interventions. Lastly, the study focused on individuals with chronic stress, and the findings may not be applicable to other populations or clinical conditions. Future research should explore the effects of tDCS and taVNS in diverse populations to broaden our understanding of their therapeutic potential.

While subjective measures provide valuable insights into individuals’ perceptions of stress and sleep quality, incorporating objective measures in future studies could enhance the robustness of findings. Objective measures, such as assessing cortisol levels to quantify physiological stress response (Adam et al., 2017) and utilizing actigraphy to objectively evaluate sleep parameters (Ancoli-Israel et al., 2003), offer complementary data to subjective reports. By integrating objective measures, researchers can validate and strengthen the evidence base for the efficacy of neuromodulatory interventions such as tDCS and taVNS in managing stress and sleep disturbances.

## 5 Conclusion

In conclusion, our study highlights the promising potential of non-invasive neuromodulation techniques, such as tDCS and taVNS, in reducing chronic stress and improving sleep quality. These interventions offer a safe, effective, and accessible approach to address the detrimental effects of stress on individuals’ wellbeing. By exploring innovative interventions that target the brain and its intricate connections with stress and sleep, we open up new possibilities for enhancing the quality of life for individuals and fostering a healthier and more resilient society. As further research unfolds, we hope to witness the continued evolution of these techniques and their integration into comprehensive healthcare approaches, ultimately benefiting both healthcare professionals and the individuals they serve.

## Data availability statement

The original contributions presented in this study are included in this article/supplementary material, further inquiries can be directed to the corresponding author.

## Ethics statement

The studies involving humans were approved by the Federal University of Alfenas Ethics Committee (CAAE 51925921.9.0000.5142) and registered in the Brazilian Registry of Clinical Trials (ReBEC) number RBR-2ww2ts8. The studies were conducted in accordance with the local legislation and institutional requirements. The participants provided their written informed consent to participate in this study.

## Author contributions

LDR: Conceptualization, Data curation, Investigation, Methodology, Writing – original draft. LPG: Conceptualization, Data curation, Investigation, Methodology, Writing – original draft. GSP: Conceptualization, Investigation, Methodology, Writing – original draft. JPSTB: Conceptualization, Data curation, Investigation, Methodology, Writing – original draft. NLC: Conceptualization, Data curation, Investigation, Methodology, Writing – original draft. MGMC: Conceptualization, Data curation, Investigation, Methodology, Writing – original draft. ROMC: Conceptualization, Data curation, Investigation, Methodology, Writing – original draft. EJRC: Conceptualization, Data curation, Investigation, Methodology, Writing – original draft. RDS: Conceptualization, Investigation, Methodology, Writing – original draft. LMAF: Conceptualization, Data curation, Funding acquisition, Investigation, Methodology, Writing – original draft. MLS: Conceptualization, Data curation, Formal analysis, Funding acquisition, Investigation, Methodology, Writing – original draft, Writing – review and editing. JRTS: Conceptualization, Data curation, Formal analysis, Funding acquisition, Investigation, Methodology, Project administration, Supervision, Writing – original draft, Writing – review and editing.
